# Using machine learning to identify local cellular properties that support re-entrant activation in patient-specific models of atrial fibrillation

**DOI:** 10.1093/europace/euaa386

**Published:** 2021-01-12

**Authors:** Cesare Corrado, Steven Williams, Caroline Roney, Gernot Plank, Mark O’Neill, Steven Niederer

**Affiliations:** 1 Department of Biomedical Engineering, King’s College London, 4th floor North Wing St Thomas’ Hospital, Westminster Bridge Road, London SE17EH, UK; 2 Division of Biophysics, Medical University of Graz, Graz, Austria

**Keywords:** Atrial fibrillation, Phase singularity map, Patient-specific model, Support vector machine

## Abstract

**Aims:**

Atrial fibrillation (AF) is sustained by re-entrant activation patterns. Ablation strategies have been proposed that target regions of tissue that may support re-entrant activation patterns. We aimed to characterize the tissue properties associated with regions that tether re-entrant activation patterns in a validated virtual patient cohort.

**Methods and results:**

Atrial fibrillation patient-specific models (seven paroxysmal and three persistent) were generated and validated against local activation time (LAT) measurements during an S1–S2 pacing protocol from the coronary sinus and high right atrium, respectively. Atrial models were stimulated with burst pacing from three locations in the proximity of each pulmonary vein to initiate re-entrant activation patterns. Five atria exhibited sustained activation patterns for at least 80 s. Models with short maximum action potential durations (APDs) were associated with sustained activation. Phase singularities were mapped across the atria sustained activation patterns. Regions with a low maximum conduction velocity (CV) were associated with tethering of phase singularities. A support vector machine (SVM) was trained on maximum local conduction velocity and action potential duration to identify regions that tether phase singularities. The SVM identified regions of tissue that could support tethering with 91% accuracy. This accuracy increased to 95% when the SVM was also trained on surface area.

**Conclusion:**

In a virtual patient cohort, local tissue properties, that can be measured (CV) or estimated (APD; using effective refractory period as a surrogate) clinically, identified regions of tissue that tether phase singularities. Combing CV and APD with atrial surface area further improved the accuracy in identifying regions that tether phase singularities.

**What’s new?**

- Left atrial size and local measurements of conduction velocity (CV) and effective refractory period (ERP) identify tissue that can sustain phase singularities.
- An support vector machine classifier can be used to identify tissue that can sustain phase singularities.
- Larger atrial size and local short action potential duration and slow CV correlates with re-entrant activation patter tethering.

## Introduction

Atrial fibrillation (AF) is a supraventricular tachyarrhythmia, characterized by unco-ordinated activation of the atria,^1^ deterioration of mechanical function,^2^ and increased incidence of cardiovascular disease, stroke, and pre-mature death.^3^ In drug-refractory patients, AF is commonly treated by radiofrequency catheter ablation (RFCA).^4^ Central to RFCA for AF is pulmonary vein isolation (PVI), where the tissue surrounding the pulmonary veins that is prone to triggered spontaneous electrical activation is electrically isolated. Pooled single-procedure 12-month arrhythmia-free survival following PVI only in paroxysmal and in persistent AF is 66.6% and 51.9%,^5^ respectively. Ablation targets beyond PVI are likely needed to improve RFCA success rates for AF patients. To improve success rates, additional lesion sets that aim to remove specific regions of tissue or substrate that support AF have been proposed. An early, and initially promising, approach for identifying AF sustaining tissue was targeting complex fractionated atrial electrograms (CFAE).^6^ However, further larger studies found that the addition of CFAE ablation did not improve patient outcomes.^7^ Subsequent re-entrant activation substrate ablation strategies have targeted focal or re-entrant activation patterns identified with basket catheters^8^ or high frequency activation regions,^9^ however, these approaches have yet to produce consistent results.

Many of the proposed ablation targets require high spatial and temporal resolution activation maps. While high-density catheter mapping systems are available, they only map from one region of the atria at a time. Catheters that map the whole atria are lower density or rely on non-contact mapping, both approaches can lead to artefacts.^10^ This can make it hard to reliably identify ablation targets. However, implicit in strategies that target tissue that support re-entrant activation, is that a combination of material properties and/or location leads to a specific region of tissue playing a critical role in sustaining the arrhythmia. We hypothesise that if re-entrant supporting tissue has specific characteristics, then these can be identified by local mapping of tissue properties.

Biophysical computer simulations of the atria provide a computational framework for linking cellular properties to emergent tissue scale properties and provide high spatial and temporal resolution signals across the entire atria for identifying re-entrant activation patterns and identifying the regions of tissue that support these activation patterns. Workflows for creating and validating models of the atria have been proposed based on CMR images or electro-anatomical maps.^11^^,^^12^ These personalized models of the atria have developed to a point where they are being used to guide therapies^13^ and virtual patient cohorts have been used to investigate patient-specific lesions, response rates, and experimental ablation procedures.^14^^,^^15^

Virtual cohorts provide a testing environment for studying the local tissue properties that support re-entrant activation patterns. We aim to create a validated virtual cohort of patient-specific atrial models that capture patient-specific electrical heterogeneity to simulate AF and then use machine learning to identify the tissue properties associated with sustained re-entrant arrhythmias in the left atria.

## Methods

In this article, we generate personalized computer models of the left atrium of a cohort of 10 patients. The model was fitted to activation measurements recorded across the atria at up to 16 sites, as described by Corrado *et al*.^12^ To trigger AF, we applied a burst pacing protocol on 12 different locations in the proximity of the pulmonary veins. On simulations that presented a self-sustaining (SS) AF, we evaluate the phase (PS) singularity map to classify the tissue capable of tether PS. Finally, we trained a SVM classifier on maximum local conduction velocity (CV_max_), and action potential duration (APD_max_) to identify portions of tissue that tether PS.

We provided a detailed description of the methods in Supplementary material online.

## Results

### Virtual patient cohort

We recruited 40 patients; 10 patients with AF had sufficient evenly spaced recording points in the left atria to allow us to create a model. These included seven paroxysmal AF (PAF) and three persistent AF (PsAF) cases. *Table 1* summarizes patient characteristics.

**Table 1:** Summary of patient characteristics

Case	Age (years)	Sex	AF duration (years)	AF class	LA diameter (cm)	LVEF (%)	Surface (cm^2^)
1	46	F	1	PAF	3.4	70	259.4
2	70	M	3	PAF	3.9	55	296.1
3	48	M	5	PAF	3.7	60	271.4
4	69	F	1	PAF	3.8	55	303.3
5	44	F	1	PAF	3.9	60	286.5
6	72	M	3	PsAF	5	60	271.9
7	71	M	3	PsAF	3.9	65	439.2
8	61	M	1	PAF	4.5	60	285.1
9	73	F	6	PAF	3.1	60	276.3
10	48	M	2	PsAF	3.2	53	299.9
	60.2 ± 12.3	60% M	2.6 ± 1.8	70% PAF	3.84 ± 0.6	60 ± 5	298.9 ± 51.2

F, female; PAF, paroxysmal AF; LVEF, left ventricular ejection fraction; M, male; PsAF, persistent AF.

### Creating and validating the model

In this article, we generate personalized computer models of the left atrium of a cohort of 10 patients. The model was fitted to activation measurements recorded across the atria at 42–100 recording sites per case, as described by Corrado et al.^12^

**Figure 1:**
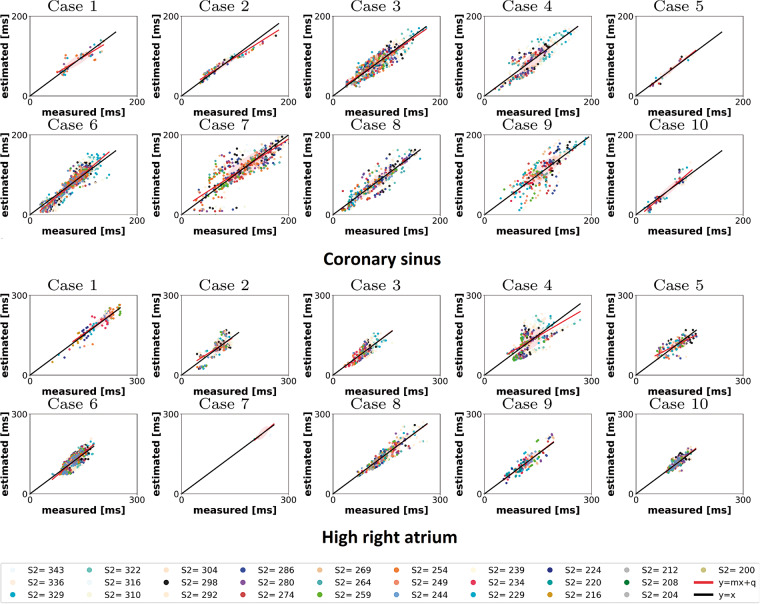
Measured vs. estimated LAT for the personalized model. Top: CS; bottom: HRA. Each colour represents a different S2 value. Each point represents a measured vs. computed LAT at each electrode, fixed S2. The black line represents a perfect match of measured and estimated LAT. The red line is the best linear fit to the measured and estimated LAT. CS, coronary sinus; HRA, high right atrium; LAT, local activation time.

Patient specific, spatially varying model parameters were fitted using an S1–S2 pacing protocol applied through the coronary sinus. Recordings were made at 14 ± 3 sites across the atria. Two model parameters (the conductivity and τ_close_) were analytically mapped to CV_max_ and APD_max_ allowing these parameters to be fitted directly to functional measurements. The remaining cellular depolarization and repolarization parameters (*h*_min_, *τ*_open_, *τ*_in_) were then fit to measured local activation times and the effective refractory period.^12^ The predicted activation times across the whole atria, when using locally fitted material properties, are compared against measured activation times used to fit the model (*Figure 1*, top). The model was validated by comparing predicted with clinically measured activation times during an S1–S2 pacing protocol applied at the high right atrium (*Figure 1*, bottom). The validation data were not used in training the models. The distribution of the fitted parameters for each patient for each parameter is presented in Supplementary material online, *Figures S1–S5*.

### Induction of atrial fibrillation and atrial tachycardia

**Figure 2:**
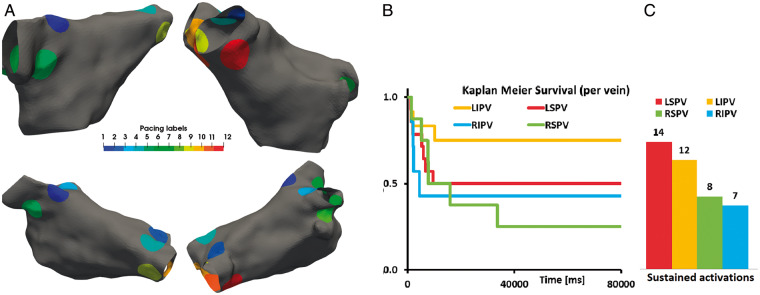
(*A*) Pacing regions used in numerical simulations and corresponding ID. (*B*) Duration sustained activations per vein. (*C*) Total number of cases presenting sustained activations for each vein.

To systematically evaluate the capacity of each atria to sustain AF, we applied a burst pacing protocol from one of 12 sites, 3 around each pulmonary vein (*Figure 2A*), and measured the type (AF, meandering rotor, or macro-re-entrant), frequency, and duration of sustained activation. Sixty-six percent of simulations terminated within 1 s and were classified as non-triggering, 8% terminated between 1 and 3 s, 9% terminated between 3 and 40 s, and 17% were classified as sustained activation and remained in activation after 80 s (*Figure 2B*). About 7.5%, 4.2%, and 5% of simulations that led to sustained AF with a frequency of 6.05 ± 0.98 Hz, meandering rotor with a frequency of 3.89 ± 0.23 Hz, or stable-re-entrant activation with a frequency of 4.77 ± 0.4 Hz, respectively.

### Transient changes in activation dynamics

Self-sustained activation in the model is initiated from a model of sinus rhythm. There is likely to be a transitory period, where initial conditions are still important, at the start of the simulation. Similarly, there may be a change in frequency prior to termination of SS activation patterns. We measured the frequency of activation in 500 ms windows. In non-terminating simulations, the activation pattern reached the activation frequency achieved in the final 5 s of simulation after a median of 303–341 ms in AF, meandering rotor and macro re-entry cases. We saw no change in the activation frequency in the window when activation terminated and the prior window. The temporal evolution of the activation frequency is plotted in Supplementary material online, *Figures S11–S14*, for each patient and for each pacing site. For our analysis, we consider activation times from 500 ms through to, whichever comes first of, termination or 80 s.

### Importance of pacing site on duration of self-sustaining activation

Pacing from different veins caused different durations of activation patterns, with pacing from the left superior PV leading to twice as many sustained activations compared with the right infer pulmonary vein (*Figure 2C*). The ability of an atria model to sustain AF cannot be determined by simulating burst pacing to induce AF from a single site. We consider all activation patterns initiated from all the pacing sites in our analysis.

### Distributions of phase singularities

**Figure 3:**
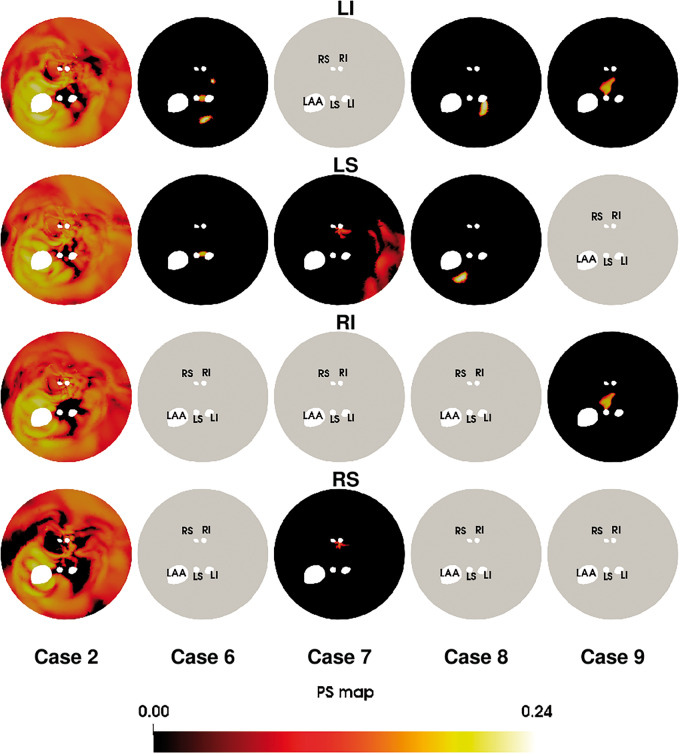
Phase singularity maps as a function of the pacing site. Each PV represents the sum of up to three maps. Grey maps indicate that no sustained activation was achieved. PV, pulmonary vein.

To test if the type of SS activation pattern supported in each atrium model was consistent, regardless of pacing location, we plotted the phase singularity maps for each SS activation pattern. This identifies if regions of tissue that tether re-entrant activation patterns were consistent, regardless of pacing site. We plot the unwrapped phase singularity density maps, starting from 75 s after pacing to remove potential transitory effects of moving rotors, for cases with sustained activation patterns (*Figure 3*). In some cases (6 and 9), the PS map is similar for different pacing sites, while for other cases (7 and 8), the PS maps are different for different pacing sites. Supplementary material online, *Figure 15* plots the combined phase singularity maps for each clinical case. There is not a unique PS map for a given atrial anatomy and set of material properties. We can only determine if a region has the potential to support a PS, not that it will support PS.

### Are specific parameter sets associated with self-sustained activation?

**Figure 4:**
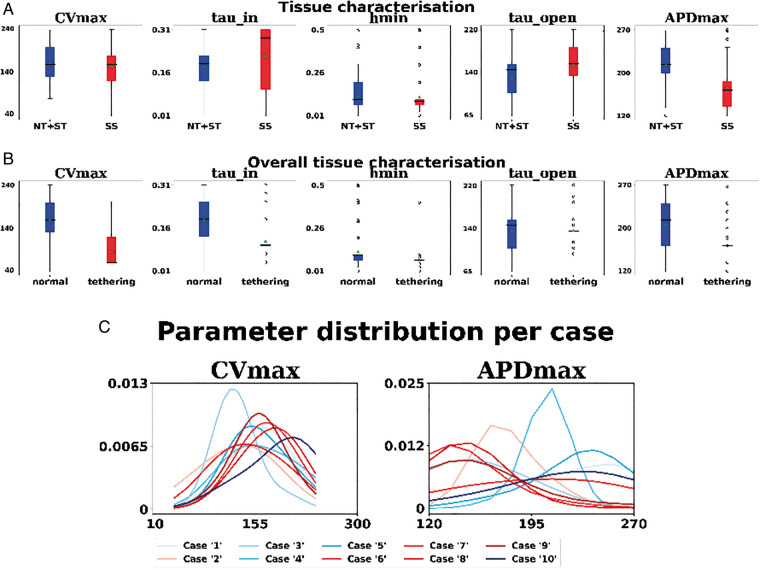
(*A*) Box plot of the parameters for cases presenting self-sustaining AF/AT (red) and either self-termination or non-triggering. (*B*) Box plot of the model parameters for a substrate with PS (red) and the other tissue (blue). Circles represent outliers. (*C*) Distribution of CV_max_ (cm^2^/s) and APD_max_ (ms) for each clinical case. Red colour scale identifies cases with self-sustaining activation pattern; blue colour scale identifies cases with self-terminating/not triggering pattern. AF, atrial fibrillation; AT, activation time.

We classified each case as self-sustaining if at least 1 of the 12 simulations produced a self-sustained activation pattern for the full 80 s and non-triggering or self-terminating (NT+ST) otherwise. We compared the distribution of fitted parameters between patients (Supplementary material online, *Figure* S*10*) and when grouped across patients (*Figure 4A*) for these two outcomes. CV_max_, *τ*_in_, *h*_min_, and *τ*_open_ have considerable overlap in parameter distribution while APD_max_ is shorter in atria with SS activation.

### Are specific parameter sets associated with phase singularities?

We classified as ‘tethering’ the portions of the tissue presenting a PS density maps at 1SD above the mean of the PS^16^ and as ‘normal’ otherwise. *Figure 4B* plots the value of the parameters that characterize the substrate that support PS (red) compared with other tissue. The CV_max_ value shows a separation between the two classes (median values: 60 cm/s on the substrate with PS; 160 cm/s in the rest of the tissue). Slower values of CV_max_ are associated with regions that anchor PS.

### How do APD_max_ and CV_max_ vary in each patient?

*Figure 4C* plots the distribution of CV_max_ and APD_max_ for each clinical case; we obtained each distribution by fitting a Gaussian Kernel and choosing the empirical standard deviation as the bandwidth. We see the high distribution of APD_max_ in four of the five cases that exhibit SS activation patterns. The majority of cases has a comparable distribution of CV_max_ regardless of their ability of the case to support a SS activation pattern. The distribution of cell model properties on all the 10 clinical cases are provided in Supplementary material online.

### Do tissue properties identify PS location?

We have demonstrated that APD_max_ and CV_max_ are correlated with SS activation and determining the location of PS. To test how well these two material properties support PS tethering, we trained a support vector machine (SVM) on the CV_max_ and APD_max_ values at every point. The SVM was trained on material properties at 3 568 549 data points, with 21 391 classified as tethering and 3 547 158 as normal.

The large data set (3 568 549 data points) and the complex correlations between the local input features (CV_max_ and APD_max_) and global features (atrium size) that produce a PS are not readily analysed using classical statistics.

The best classifier achieves an accuracy of 91% (sensitivity = 0.96, specificity = 0.91) indicating that APD_max_ and CV_max_ play an important role in the location of PS tethering.

Does atrial size impact the importance of CV_max_ and APD_max_ on PS Location?

We have previously shown that effective size, which combines an APD surrogate (ERP), CV, and area values, predicts response to PVI in persistent AF cases.^17^ To test if atrial size impacts the regions that can support PS, we retrained the SVM including each patients atrial size as an input feature. We added atrial size into the CV_max_ and APD_max_ SVMs. When atrial surface area CV_max_ and APD_max_ are taken into account, the classifier achieves an improved accuracy of 95% (sensitivity = 0.99, specificity = 0.95).

## Discussion

We have created a validated virtual cohort of 10 (7 paroxysmal and 3 persistent) AF patients with heterogeneous material properties fitted to each individual patient’s electrophysiology measurements. We show that the capacity to initiate an arrhythmia is dependent on the pacing site, after a transitory phase the activation rates remain stable, termination can still occur up to 40 s after initiation, PS maps are not unique for a given atrium anatomy and material properties, shorter APD_max_ is associated with self-sustained activations, the CV_max_ is associated with locations that support PS, that a combination of APD_max_ and CV_max_ can identify PS tethering locations with 91% accuracy and that accounting for atria size improves the identification of PS tethering locations to 95%.

### Initiating and analysing simulated atrial fibrillation

To reduce computational cost, atrial arrhythmia simulations have also adopted relatively short simulation durations, often as low as 5 s^13^ for identifying sustained arrhythmia. Here we show that 9% of self-terminations occur after 3 s and self-terminations can still occur even 40 s after initiation, motivating the use of longer simulations for clinically meaningful durations. However, 80 s is a long time to perform a simulation. For the 120 simulations that we performed, only one case would have been misclassified as self-sustained if we had adopted a 30 s simulation duration, in line with the duration used for defining an episode of AF,^18^ and this may represent a reasonable balance of clinical relevance and computational tractability.

We considered that the activation patterns may organise or slow down prior to termination. However, consistent with clinical studies,^19^^,^^20^ we found that there was no change in frequency in the models prior to terminations. We did, however, observe a transitory period following initialization of AF of ∼330 ms. This may be an underestimate of the transition period in clinical studies and simulations studies using complex models, where slower changes in ionic homoeostasis, which are not accounted for in the mMS model, may cause a longer transitory period.

### Re-entrant and ectopically sustained activation

Consistent with clinical observations that ectopic activations start from the left superior pulmonary veins,^21^ we find that pacing from the left superior pulmonary veins leads to more sustained activation patterns. In our models, only 50% of the virtual cohort cases could support a self-sustained activation pattern. We did not account for sustained ectopic events following the initial burst pacing in the models, as we aimed to only simulate the effect of the electrophysiological substrate to support re-entrant activation patterns. In many cases, particularly in paroxysmal atria, AF is initiated and potentially driven by ectopic beats, often originating in the ostium of the pulmonary veins.^21^ The inability of our atrium models to sustain AF may represent cases where their clinical AF was supported by ectopic beats and not an arrhythmogenic substrate. These patients may represent cases where PVI is more likely to be effective.

At the same time, the ability to sustain AF may be highly dependent on attributes not included in the models such as endocardial and epicardial dissociation, fibrosis, or the right atrium. We did not have access to AF induction studies in these cases, the location of ablation lesions delivered, or ablation outcome. The addition of both more sophisticated models and additional therapy and outcome data from patients would allow us to test what factors contribute to simulated sustained activation and if cases that cannot sustain activation have AF driven by pulmonary vein ectopic beats.

### Local tissue properties that support phase singularities

Previously re-entrant activation patterns have been associated local fibrotic regions measured using MRI in simulation^22^^,^^23^ and clinical^24^ studies, however, others found no link between re-entrant activation and fibrosis.^25^ Alternately, other groups have suggested regions with low voltage and CV,^26^ or regions with CFAE^27^ could support re-entrant activation. In this paper, we assumed the effects of fibrosis would be captured in electrical measurements^28^ and consequently be encoded in the fitted cell models. The classifier identified tissue associated with tethered PS with 91% accuracy and comparable F1 score. Experimental noise may impact the SVM classifier. As we trained and tested the SVM classifier using model parameters fitted to experimental data that were subject to noise, it is not expected that the accuracy of the SVM will degrade when applied to measurements with a similar level of noise. To estimate how increased noise may impact the classifier, we added a 10% white Gaussian noise into testing data set. This elevated level of noise, beyond what is seen clinically caused the accuracy of the classifier to decrease from 95% to 93%.

We also showed how patient attributes beyond tissue properties can be used to improve identification of PS tethering locations. The addition of patient atrial size further increased the ability of the SVM to identify PS tethering sites to 95% accuracy. This is consistent with many previous imaging studies that have identified atrial size or shape as potential contributors; these are used as indexes of risk, although not as a factor in guiding therapy.

### Model critique

We have created a model that reflects the complexity of the available clinical data, we have aimed to capture the data that are available in the catheter lab. This represents a trade-off between model personalization and sophistication. In our simulations, we have not attempted to model fibre distribution, biophysical cellular physiology, endo–epicardial dissociation, fibrosis, the right atria, or wall thickness. We have assumed that the effects of tissue change due to fibrosis, remodelling, or physiological heterogeneity will be captured by the measured activation times and will be encoded within the spatially varying cell model parameter values. We have not included the right atria as the source of AF is predominantly found in the left atria and the right atria is not routinely mapped during ablation procedures. While we recognise that many of the attributes we have not included in the model may play a role in AF, we were able to train and validate our model on an independent data set and did not find any evidence that we needed to include these extra attributes to replicate the clinical observations. This approach represents an alternate strategy for patient-specific atrial models from purely image-based approaches, where cellular properties are assumed common between patients and are based on MRI image intensity values.

We created and validated our model using a systematic S1–S2 pacing protocol. We did not validate the model against patient-specific electrical recordings of AF data. We do not have AF recordings for these patients. Validating patient-specific simulations of AF is challenging. Boyle *et al.*^13^ have indirectly validated their model by showing that in 10 cases the model can identify critical ablation sites. Comparison of simulated PS maps with clinical measurements is challenging due to disagreement on the number of PS locations that should be identified,^9^^,^^29^ recording devices have limited coverage and the use of open and proprietary PS identification tools. We do not see one standard PS distribution in our simulations. We see high densities of PS, consistent with^29^ (Case 2), meandering rotors, consistent with^30^ (Cases 7 and 9), and stable rotors consistent with^8^ (Cases 6 and 8). In this paper, we presented a limited number of cases as each personalized model requires a high-quality data set. Increasing the number of cases modelled, in particular for persistent AF or cases with a sustained AF, may provide a promising approach for identifying the mechanisms underpinning these different re-entrant activation patterns.

### Limitations

We modelled the atria as a shell with homogenous thickness. Some of the impacts of heterogeneous wall thickness on local conduction velocity will be captured by regional variations in conductivity in the model. However, the model we have does not capture three potentially important impacts of wall thickness on atrial electrophysiology modelling. First, gradients in wall thickness will lead thicker tissue activating thinner tissue, or vice versa, depending on the wave direction. This will lead to direction-dependent electronic loading differences that will have activation wave direction-dependent effects on the conduction velocity that cannot be captured by our proposed modelling framework. Experiments in sheep indicated that gradients in wall thickness may impact the tethering of re-entrant activation patterns.^31^ However, simulations in human left atrium, including wall thickness, did not find an impact of wall thickness gradients on re-entrant activation tethering in the left atrium.^23^ Second, wall thickness raises the potential for distinct activation patterns to occur on the endocardium and epicardium, this would explain increased complex fractionated electrograms in thicker tissue.^32^ Different activation waves on endocardial and epicardial tissue may lead to measurement artefacts that compromise the fitted model parameters. Third, our model cannot capture transmural differences in fibrosis, which simulation studies have shown could play a role in AF activation patterns.^33^

## Conclusions

We have created a virtual patient cohort encoding patient-specific electrophysiology heterogeneity. We have used the models combined with machine learning to show that combining local CV and APD with left atrial size can be used to identify tissue that can sustain phase singularities. Using a mathematical model that captures patient-specific dynamic atrial electrophysiology response we found that larger left atrium atrial size and local short APD and slow CV were correlated with re-entrant activation patter tethering. This is consistent with previous studies, supporting the critical mass hypothesis for sustain AF, that have correlated left atrium size and measures of left atrium wave length and ERP with AF.^17^^,^^34^^,^^35^ Importantly our study indicates that it is not only the average but also the local CV and APD within the context of atrial size that determines the ability of an atria to tether re-entrant activation patterns. This may provide an alternate method for identifying ablation targets or guiding anti-arrhythmic drug therapies that adapt CV and APD, to remove these regions.

## Supplementary material

Supplementary material is available at *Europace* online.

## Funding

This work was supported by the British Heart Foundation (PG/13/37/30280) and the Department of Health via the National Institute for Health Research (NIHR) comprehensive Biomedical Research Centre award to Guy’s & St Thomas’ NHS Foundation Trust in partnership with King’s College London and King’s College Hospital NHS Foundation Trust. This paper is part of a supplement supported by an unrestricted grant from the Theo-Rossi di Montelera (TRM) foundation.

**Conflict of interest:** none declared. 

## Data availability

The data underlying this article cannot be shared publicly due to privacy reasons. The data will be shared on reasonable request to the corresponding author.

## Supplementary Material

euaa386_Supplementary_Data

## References

[1] Skanes AC , Mandapati R, Berenfeld O, Davidenko JM, Jalife J. Spatiotemporal periodicity during atrial fibrillation in the isolated sheep heart. Circulation 1998;98:1236–48. 9743516 10.1161/01.cir.98.12.1236

[2] Reant P , Lafitte S, JaïS P, Serri K, Weerasooriya R, Hocini M et al Reverse remodeling of the left cardiac chambers after catheter ablation after 1 year in a series of patients with isolated atrial fibrillation. Circulation 2005;112:2896–903. 16260634 10.1161/CIRCULATIONAHA.104.523928

[3] Sumeet SC , Rasmus H, Kumar N, David S, Michiel R, Emelia JB et al Worldwide epidemiology of atrial fibrillation. Circulation 2014;129:837–47. 24345399 10.1161/CIRCULATIONAHA.113.005119 PMC4151302

[4] HaïSsaguerre M , Shah DC, JaïS P, Hocini M, Yamane T, Deisenhofer I et al Electrophysiological breakthroughs from the left atrium to the pulmonary veins. Circulation 2000;102:2463–5. 11076817 10.1161/01.cir.102.20.2463

[5] Ganesan AN , Shipp NJ, Brooks AG, Kuklik P, Lau DH, Lim HS et al Long-term outcomes of catheter ablation of atrial fibrillation: a systematic review and meta-analysis. JAHA 2013;2:e004549. 23537812 10.1161/JAHA.112.004549 PMC3647286

[6] Verma A , Novak P, Macle L, Whaley B, Beardsall M, Wulffhart Z et al A prospective, multicenter evaluation of ablating complex fractionated electrograms (CFEs) during atrial fibrillation (AF) identified by an automated mapping algorithm: acute effects on AF and efficacy as an adjuvant strategy. Heart Rhythm 2008;5:198–205. 18242539 10.1016/j.hrthm.2007.09.027

[7] Verma A , Jiang C-y, Betts TR, Chen J, Deisenhofer I, Mantovan R et al Approaches to catheter ablation for persistent atrial fibrillation. N Engl J Med 2015;372:1812–22. 25946280 10.1056/NEJMoa1408288

[8] Narayan SM , Krummen DE, Shivkumar K, Clopton P, Rappel W-J, Miller JM. Treatment of atrial fibrillation by the ablation of localized sources. CONFIRM (Conventional Ablation for Atrial Fibrillation With or Without Focal Impulse and Rotor Modulation). Trial 2012;60:628–36. 10.1016/j.jacc.2012.05.022 PMC3416917 22818076

[9] Atienza F , Almendral J, Ormaetxe JM, Moya Á, Martínez-Alday JD, Hernández-Madrid A et al Comparison of radiofrequency catheter ablation of drivers and circumferential pulmonary vein isolation in atrial fibrillation: a noninferiority randomized multicenter RADAR-AF trial J Am Coll Cardiol 2014;64:2455–67. 25500229 10.1016/j.jacc.2014.09.053

[10] Roney CH , Cantwell CD, Bayer JD, Qureshi NA, Lim PB, Tweedy JH et al Spatial resolution requirements for accurate identification of drivers of atrial fibrillation. Circ Arrhythm Electrophysiol 2017;10:e004899. 28500175 10.1161/CIRCEP.116.004899 PMC5434962

[11] Corrado C , Razeghi O, Roney C, Coveney S, Williams S, Sim I et al Quantifying atrial anatomy uncertainty from clinical data and its impact on electro-physiology simulation predictions. Med Image Anal 2020;61:101626. 32000114 10.1016/j.media.2019.101626

[12] Corrado C , Williams S, Karim R, Plank G, O’Neill M, Niederer S. A work flow to build and validate patient specific left atrium electrophysiology models from catheter measurements. Med Image Anal 2018;47:153–63. 29753180 10.1016/j.media.2018.04.005 PMC5998385

[13] Boyle PM , Zghaib T, Zahid S, Ali RL, Deng D, Franceschi WH et al Computationally guided personalized targeted ablation of persistent atrial fibrillation. Nat Biomed Eng 2019;3:870–9. 31427780 10.1038/s41551-019-0437-9 PMC6842421

[14] Roney CH , Beach ML, Mehta A, Sim I, Corrado C, Bendikas R et al In silico comparison of left atrial ablation techniques that target the anatomical, structural and electrical substrates of atrial fibrillation. Front Physiol 2020;11:1145. 33041850 10.3389/fphys.2020.572874 PMC7526475

[15] Roney CH , Williams SE, Cochet H, Mukherjee RK, O’Neill L, Sim I et al Patient-specific simulations predict efficacy of ablation of interatrial connections for treatment of persistent atrial fibrillation. Europace 2018;20:iii55–68. 30476055 10.1093/europace/euy232 PMC6251187

[16] Roney C , Beach M, Mehta A, Sim I, Corrado C, Bendikas R et al In silico comparison of left atrial ablation techniques that target the anatomical, structural and electrical substrates of atrial fibrillation. Front Physiol 2020;11:1145. https://www.frontiersin.org/articles/10.3389/fphys.2020.572874/full) 33041850 10.3389/fphys.2020.572874 PMC7526475

[17] Williams SE , O’Neill L, Roney CH, Julia J, Metzner A, Reißmann B et al Left atrial effective conducting size predicts atrial fibrillation vulnerability in persistent but not paroxysmal atrial fibrillation. J Cardiovasc Electrophysiol 2019;30:1416–27. 31111557 10.1111/jce.13990 PMC6746623

[18] Calkins H , Hindricks G, Cappato R, Kim Y-H, Saad EB, Aguinaga L et al; Document Reviewers. 2017 HRS/EHRA/ECAS/APHRS/SOLAECE expert consensus statement on catheter and surgical ablation of atrial fibrillation. Europace 2018;20:e1–160. 10.1093/europace/eux274 PMC5834122 29016840

[19] Bollmann A , Kanuru N, McTeague K, Walter P, DeLurgio D, Langberg J. Frequency analysis of human atrial fibrillation using the surface electrocardiogram and its response to ibutilide. Am J Cardiol 1998;81:1439–45. 9645894 10.1016/s0002-9149(98)00210-0

[20] Fujiki A , Sakabe M, Nishida K, Mizumaki K, Inoue H. Role of fibrillation cycle length in spontaneous and drug-induced termination of human atrial fibrillation. Circ J 2003;67:391–5. 12736475 10.1253/circj.67.391

[21] Haïssaguerre M , Jaïs P, Shah DC, Takahashi A, Hocini M, Quiniou G et al Spontaneous initiation of atrial fibrillation by ectopic beats originating in the pulmonary veins. N Engl J Med 1998;339:659–66. 9725923 10.1056/NEJM199809033391003

[22] Deng D , Murphy MJ, Hakim JB, Franceschi WH, Zahid S, Pashakhanloo F et al Sensitivity of reentrant driver localization to electrophysiological parameter variability in image-based computational models of persistent atrial fibrillation sustained by a fibrotic substrate. Chaos 2017;27:093932. 28964164 10.1063/1.5003340 PMC5605332

[23] Roy A , Varela M, Aslanidi O. Image-based computational evaluation of the effects of atrial wall thickness and fibrosis on re-entrant drivers for atrial fibrillation. Front Physiol 2018;9:1352. https://www.frontiersin.org/articles/10.3389/fphys.2018.01352/full) 30349483 10.3389/fphys.2018.01352 PMC6187302

[24] Cochet H , Dubois R, Yamashita S, Al Jefairi N, Berte B, Sellal J-M et al Relationship between fibrosis detected on late gadolinium-enhanced cardiac magnetic resonance and re-entrant activity assessed with electrocardiographic imaging in human persistent atrial fibrillation. JACC: Clin Electrophysiol 2018;4:17–29. 29479568 10.1016/j.jacep.2017.07.019 PMC5824731

[25] Chrispin J , Gucuk Ipek E, Zahid S, Prakosa A, Habibi M, Spragg D et al Lack of regional association between atrial late gadolinium enhancement on cardiac magnetic resonance and atrial fibrillation rotors. Heart Rhythm 2016;13:654–60. 26569460 10.1016/j.hrthm.2015.11.011

[26] Honarbakhsh S , Schilling RJ, Orini M, Providencia R, Keating E, Finlay M et al Structural remodeling and conduction velocity dynamics in the human left atrium: relationship with reentrant mechanisms sustaining atrial fibrillation. Heart Rhythm 2019;16:18–25. 30026014 10.1016/j.hrthm.2018.07.019 PMC6317307

[27] Ammar-Busch S , Reents T, Knecht S, Rostock T, Arentz T, Duytschaever M et al Correlation between atrial fibrillation driver locations and complex fractionated atrial electrograms in patients with persistent atrial fibrillation. Pacing Clin Electrophysiol 2018;41:1279–85. 30133719 10.1111/pace.13483

[28] Fukumoto K , Habibi M, Ipek EG, Zahid S, Khurram IM, Zimmerman SL et al Association of left atrial local conduction velocity with late gadolinium enhancement on cardiac magnetic resonance in patients with atrial fibrillation. Circ Arrhythm Electrophysiol 2016;9:e002897. 26917814 10.1161/CIRCEP.115.002897 PMC4772170

[29] Child N , Clayton RH, Roney CH, Laughner JI, Shuros A, Neuzil P et al Unraveling the underlying arrhythmia mechanism in persistent atrial fibrillation: results from the STARLIGHT study. Circ Arrhythm Electrophysiol 2018;11:e005897. 29858382 10.1161/CIRCEP.117.005897

[30] Haissaguerre M , Hocini M, Denis A, Shah AJ, Komatsu Y, Yamashita S et al Driver domains in persistent atrial fibrillation. Circulation 2014;130:530–8. 25028391 10.1161/CIRCULATIONAHA.113.005421

[31] Yamazaki M , Mironov S, Taravant C, Brec J, Vaquero LM, Bandaru K et al Heterogeneous atrial wall thickness and stretch promote scroll waves anchoring during atrial fibrillation. Cardiovasc Res 2012;94:48–57. 22227155 10.1093/cvr/cvr357 PMC3307378

[32] Wi J , Lee H-J, Uhm J-S, Kim J-Y, Pak H-N, Lee M et al Complex fractionated atrial electrograms related to left atrial wall thickness. J Cardiovasc Electrophysiol 2014;25:1141–9. 24948440 10.1111/jce.12473

[33] Gharaviri A , Bidar E, Potse M, Zeemering S, Verheule S, Pezzuto S et al Epicardial fibrosis explains increased endo–epicardial dissociation and epicardial breakthroughs in human atrial fibrillation. Front Physiol 2020;11: 68.https://www.frontiersin.org/articles/10.3389/fphys.2020.00068/full) 32153419 10.3389/fphys.2020.00068 PMC7047215

[34] Hwang M , Park J, Lee Y, Park JH, Choi SH, Shim EB et al Fibrillation number based on wavelength and critical mass in patients who underwent radiofrequency catheter ablation for atrial fibrillation. IEEE Trans Biomed Eng 2015;62:673–9. 25343755 10.1109/TBME.2014.2363669

[35] Byrd GD , Prasad SM, Ripplinger CM, Cassilly TR, Schuessler RB, Boineau JP et al Importance of geometry and refractory period in sustaining atrial fibrillation. Circulation 2005;112:I-7–13. 16159868 10.1161/CIRCULATIONAHA.104.526210

