# Supplementary material for: Using machine learning to identify local cellular properties that support re-entrant activation in patient-specific models of atrial fibrillation
Source: Europace. 2021 Jan 12;23(Suppl 1):i12–20. doi: 10.1093/europace/euaa386 (PMC7943361; doi:10.1093/europace/euaa386)
Supplement: euaa386_Supplementary_Data [file euaa386_supplementary_data.docx]

Using Machine Learning to Identify Local Cellular Properties that Support Re-entrant Activation in Patient Specific Models of Atrial Fibrillation

# Online Supplement

# Extended Methods

## Patient selection and clinical procedure

In this paper, we considered clinical patients suffering from atrial fibrillation (AF) and undergoing first time radio frequency catheter ablation. Ethical approval was granted by the National Research Ethics Service (10/H0802/77) and all participants gave written informed consent for inclusion in the study. The research conformed to the principles described in the Declaration of Helsinki. Patients with ischemic heart disease, cardiac surgery or structural heart disease were excluded. Anti-arrhythmic drugs, including calcium channel blockers, were stopped at least 5 half-lives before ablation. Amiodarone was stopped at least 6 weeks prior to ablation. All clinical procedures were performed under general anaesthesia. Following femoral access and trans-septal puncture, two 8.5 French SR0 long sheaths and a PentaRay mapping catheter (Biosense Webster, CA, 1mm electrode size, 4-4-4mm spacing) were advanced into the LA. Decapole (St Jude Medical, MN) and pentapole (Bard Electrophysiology, MA) catheters were positioned in the coronary sinus (CS) and high right atrium (HRA), respectively.

## Pacing protocol

The pacing protocol was delivered using a custom-built, institutionally-approved [stimulator](https://www.sciencedirect.com/topics/engineering/stimulator) and consisted of a 2-beat drive train (S1 = 470 ms) followed by a single premature extra stimulus S2<S1. The S1-S2 coupling interval was reduced continuously and without operator interference in 2% steps from 343 ms to either 200 ms or loss of capture. All pacing stimuli were delivered at a voltage of at least twice the [threshold voltage](https://www.sciencedirect.com/topics/computer-science/threshold-voltage) and with a pulse width of 2 ms. Pacing stimuli were delivered from either the CS or the HRA, while the activation times were recorded in the body of the LA using a PentaRay catheter. The PentaRay catheter was sequentially manoeuvred to multiple sites on the [endocardial](https://www.sciencedirect.com/topics/medicine-and-dentistry/endocardium) surface of the LA; bipolar electrograms were recorded throughout in response to complete S1-S2 pacing trains, delivered from the mid-CS or HRA.

## Processing the electrograms

We processed the atrial bipolar electrograms from the PentaRay using an in-house developed MATLAB graphical user interface (GUI https://www.cardiacsoftwarepartners.org/). This interface reads in the output files from the electroanatomical mapping system (Ensite Velocity St Jude) and registers the positions of the recording electrodes to the mapped atrial geometry. For each bipolar pair of electrodes and for each S1-S2 coupling interval, we evaluated LATs as the time that the first peak (or valley) on the electrogram trace occurs. On [bipolar electrodes](https://www.sciencedirect.com/topics/engineering/bipolar-electrode), where measurements are available, we evaluated CV using a [piecewise](https://www.sciencedirect.com/topics/engineering/piecewise) linear approach, similar to that described in (Cantwell, Roney et al. 2015) and summarised below as follows:

1. On the site identified by the electrodes forming the PentaRay catheter, we interpolate the LATs measured at the 10 bipolar electrodes using piecewise-linear polynomia and a Delaunay [triangulation](https://www.sciencedirect.com/topics/computer-science/triangulation).
2. We locally compute the gradient of the interpolated LATs and then we evaluate the modulus of the local CV as the inverse of the modulus of the gradient.
3. On each electrode that captured a signal and for each premature extra stimulus S2, we identified the local CV as the median of the computed CV values within a circular region of radius R=2.5 cm around the electrode. This radius corresponds to twice of the length of a catheter spline and removes spurious CV values from the fitting.

All calculated fields (LAT and CV at each electrode for each pacing protocol) and the anatomy are then exported to [VTK](https://www.sciencedirect.com/topics/medicine-and-dentistry/vtk) files (Schroeder, Lorensen et al. 2004) providing the input for the model personalisation process.

## Generating the input for the fitting algorithm

From the local CV values, described above, we generated local CV restitutions using the PentaRay electrograms at each electrode and for each S1-S2 pacing protocol when the atrium was paced in the CS. CV values were defined to be outside of the physiological range and were excluded from subsequent analysis if they were greater than 200 cm/s. We determined this value by first plotting the CV distribution for all the S2 coupling intervals and all the clinical data considered, then fitting that distribution to a normal distribution and rounding the value that corresponds to the mean+2SD. The distribution in CV is consistent with those reported in (Okano, Igarashi et al. 2010); (Weber, Luik et al. 2011) on the left atrium and with those reported in (Schilling, Peters et al. 2001) for the right atrium. We estimated the local ERP value as the largest s_2_ interval that did not produce a local activation at the PentaRay electrode. This value was termed S1-S2_block_ in (Williams, Linton et al. 2017). Since the pacing protocol uses a single s_1_ value, only one ERP value is estimated at each PentaRay electrode, not a restitution curve.

## Creation and Validation of Virtual Patient Cohort

For each patient, we generate a computational model with the workflow described in (Corrado, Williams et al. 2018) and sketched in Figure 1.


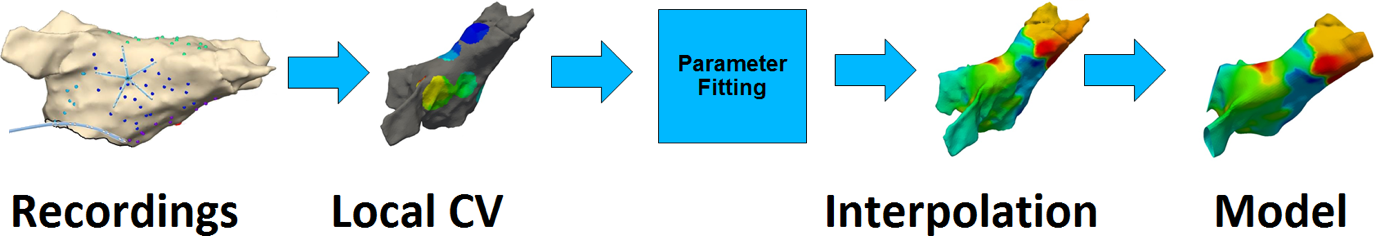


Figure 1 Sketch of the workflow used to generate a patient-specific computational model. We first collect local EGM, then we evaluate LAT and local CV restitutions following a programmed pacing at CS. Next, we fit the model parameter, we interpolate them using a nearest neighbour criterion. Finally, we generate a computational mesh.

First, we collected local electrogram recordings (EGM) by manoeuvring a Penta Ray catheter on the left atrium epicardium and applying the S1-S2 pacing protocol. We paced from a remote catheter located in the coronary sinus (CS). Next, we evaluated local activation times (LATs) and local conduction velocity (CV) restitution and the local functional block as a surrogate of the local effective refractory period (ERP). We fitted the local parameter values of the modified Mitchell and Schaeffer (mMS) ionic model (Corrado and Niederer 2016) following the method we published previously (Corrado, Whitaker et al. 2016, Corrado, Whitaker et al. 2017). Finally, we interpolated local parameters using the nearest neighbour criterion.

The mesh from the electro anatomical mapping system was refined to achieve a regular triangulated mesh with a mean edge length of ~200 μm for simulation. For all whole atria simulations we solved the monodomain on this mesh with a time step of 25 μs for the diffusive problem and 2.5 μs for the ionic model.

We validated the workflow with the procedure described in (Corrado, Williams et al. 2018) using a separate validation data set of LAT measured on the LA endocardium during an S1-S2 pacing protocol from a remote catheter located in the high right atrium (HRA).

Figure 2 sketches the procedure used to validate the workflow.


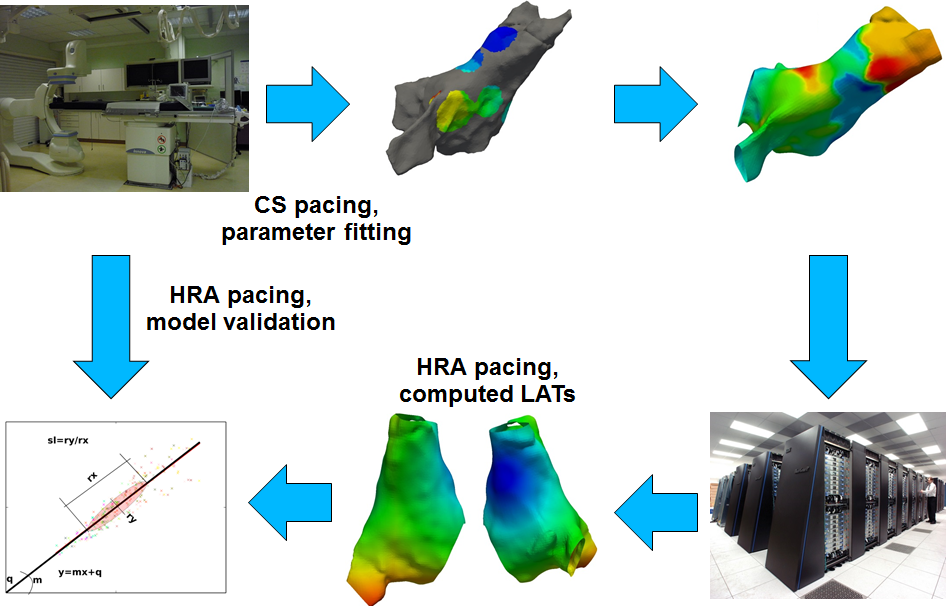


Figure 2 Sketch of the procedure used to validate the workflow. We build a personalised computer model of the left atrium from a set of LATs obtained pacing on the CS. Next, we compute numerical simulations of a programmed pacing protocol with a stimulus applied on the HRA. Finally, we compare the measured and the simulated LAT at catheter electrodes.

## Simulation of the atrial fibrillation

To initiate AF we applied a stimulus in the proximity of each pulmonary vein on a circular region of radius R=1cm. For each pulmonary vein, we followed this procedure for 3 evenly-spaced circular regions, manually chosen on each clinical case. Hence, for each clinical case, we computed 12 simulations. Figure 3 shows all the 12 regions and the corresponding label.

To attempt to initiate AF we applied a burst pacing protocol form each one of these pacing sites separately. We started pacing from an initial cycle length (CL) of 200 ms; for each CL, we applied 5 consecutive stimuli and then we decremented the CL by 5ms. We repeated the procedure down to a CL of 100 ms. This AF triggering protocol is routinely used during clinical procedures (Williams, O’Neill et al. 2019).


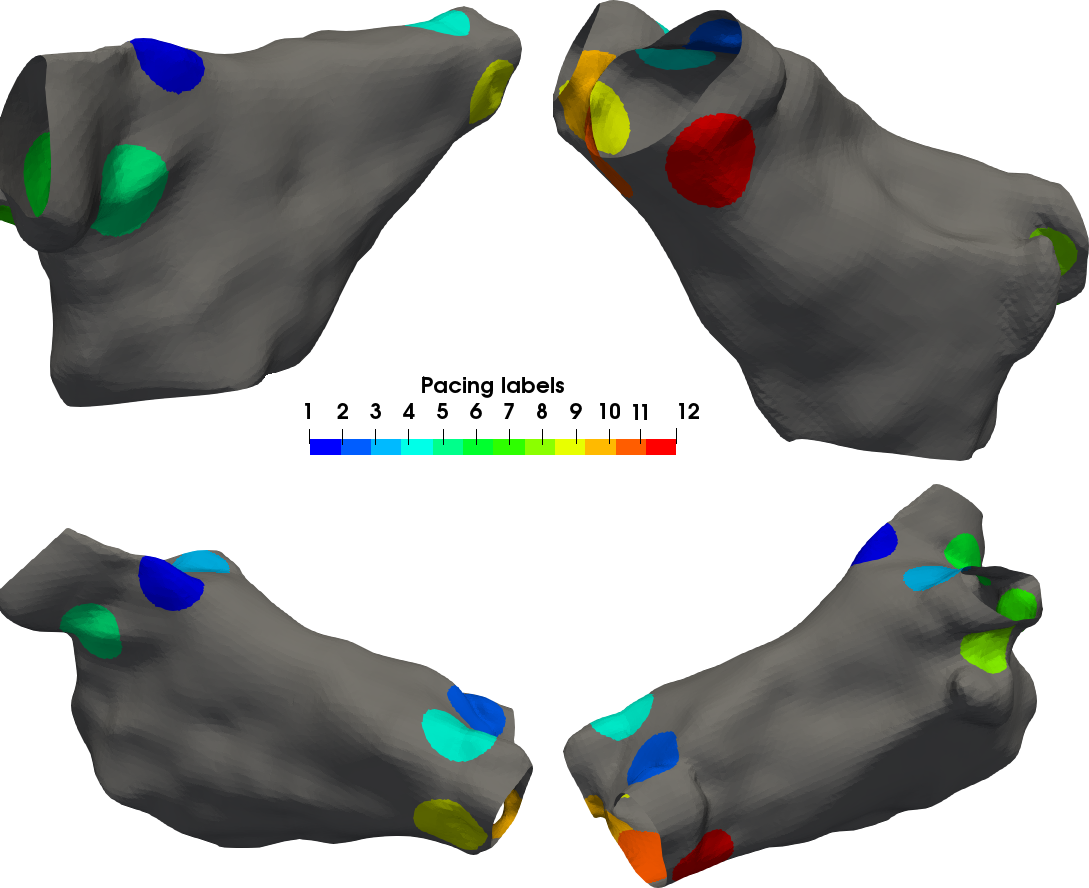


Figure 3 Pacing regions used in numerical simulations and corresponding ID. For each PV, we applied the tested 3 different evenly-spaced pacing locations

We simulated the electrical activity for up to 80 seconds after the application of the programmed pacing and we measured the local activation times as the time where the trans-membrane potential rose over 70% of its maximum amplitude (this value corresponds to 0.7 in the dimensionless mMS ionic model) and with positive time derivative.

## Outcome analysis

We classified the solution of each simulation as follows:
*Non-Triggering AF/AT*. No spontaneous activations are present 1 second behind the pacing procedure. In this case, a focus located in the stimulus region cannot trigger/sustain AF.

*Self-terminating AF/AT.* Spontaneous activations are present but last no more than 75 seconds. In this case, ectopic foci may sustain an AF. When we detect a self-terminating AF, we determine the time elapsed to the termination.

*Self-sustaining AF/AT.* Self-sustaining activations are present at 80 seconds following the pacing procedure. This classification includes both atrial fibrillation and atrial tachycardia and we computed the phase singularity map for these simulations, following the procedure described in (Rogers 2004) and averaging on the last 5 simulated seconds.

We evaluated the time evolution of the local activation rate on the simulations that presented either a self-terminating or self-sustaining activation pattern. We proceeded as follows. For each mesh point and for each pair of subsequent activations $\left( T_{i-1},T_{i} \right)$ we generated the time, frequency pair $\left( t_{i},f_{i} \right)$where $t_{i}=\left( T_{i}+T_{i-1} \right)/2$ and$f_{i}=1/\left( T_{i}-T_{i-1} \right)$. Next, we ordered the samples $\left( t_{i},f_{i} \right)$with respect to $t_{i}$ in ascending order. Next, we divided the temporal axis into 500 ms windows and finally, we evaluated the mean and the standard deviation of $f_{i}$ within each 500 ms window. This provides a temporal evolution of the global activation rate.

## Tissue Classification

For each clinical case, we computed the global PS map by collecting all the PS maps on the patient left atrium; whenever 2 or more non-zero PS regions were superimposed, we assigned the largest value on the region where they superimposed. For each patient, we then identified the portions of the tissue that can tether an arrhythmia (PS hotspots) by thresholding the PS density maps at 1 standard deviation above the mean (Roney, Beach et al. 2020); the portions of tissue presenting a value of the PS map largest than the threshold are classified as “tethering” and “non-tethering” otherwise. We further classified as “non-tethering” the whole tissue of cases not presenting a self-sustaining pattern as any regions that caused periods of tethering in these cases were insufficient to lead to a sustained activation. Next, we collected the parameters associated with “tethering” and “non-tethering” regions into 2 parameter sets.

We trained an SVM classifier to differentiate the two parameter sets. We trained the SVM on 3568549 data points, with 21391 classified as tethering and 3547158 as normal. We evaluated SVM accuracy with a 5-fold cross-validation test; to account for the imbalanced data set we divided each endpoint set into training and test sets (80% training, 20% test), hence preserving the same endpoint proportions on the training and the test sets. Before training the SVM classifier, for each fold we centred the input data on the mean of the training set and scaled by the standard deviation. We evaluate the accuracy as the mean accuracy over the 5 folds on the test set. We then evaluated the precision, the recall, the F1 score and the confusion matrix on the entire set of samples (training + test) and averaged over the 5 trained classifiers.

# Results

## Model properties

Following the fitting and interpolation of model properties, we generate maps of model parameters across the atria. We can view the distribution of these parameters on an unfolded anatomy obtained using the algorithm described in (Karim, Ma et al. 2014). Figure 4 plots the map of the maximum conduction velocity, Figure 5 the map of the maximum action potential duration, Figure 6 the map of τ_open_, Figure 7 the map of h_min_ and Figure 8 the map of τ_in_.


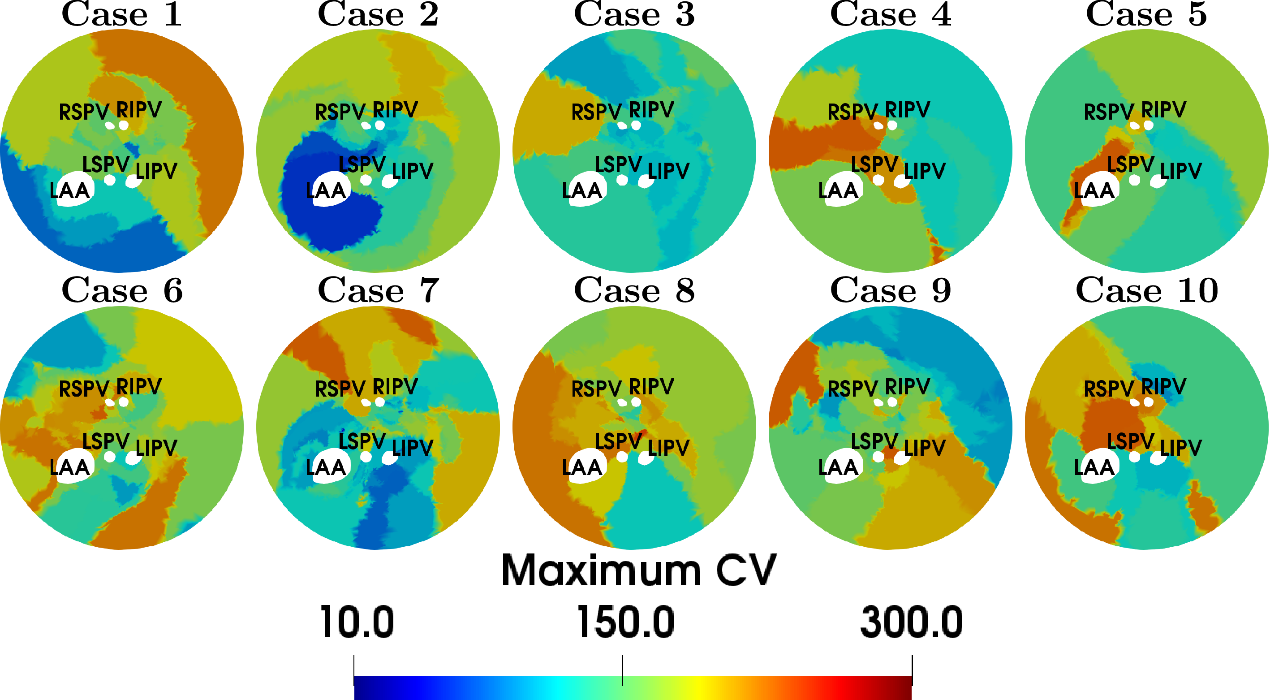


Figure 4 Map of the maximum conduction velocity


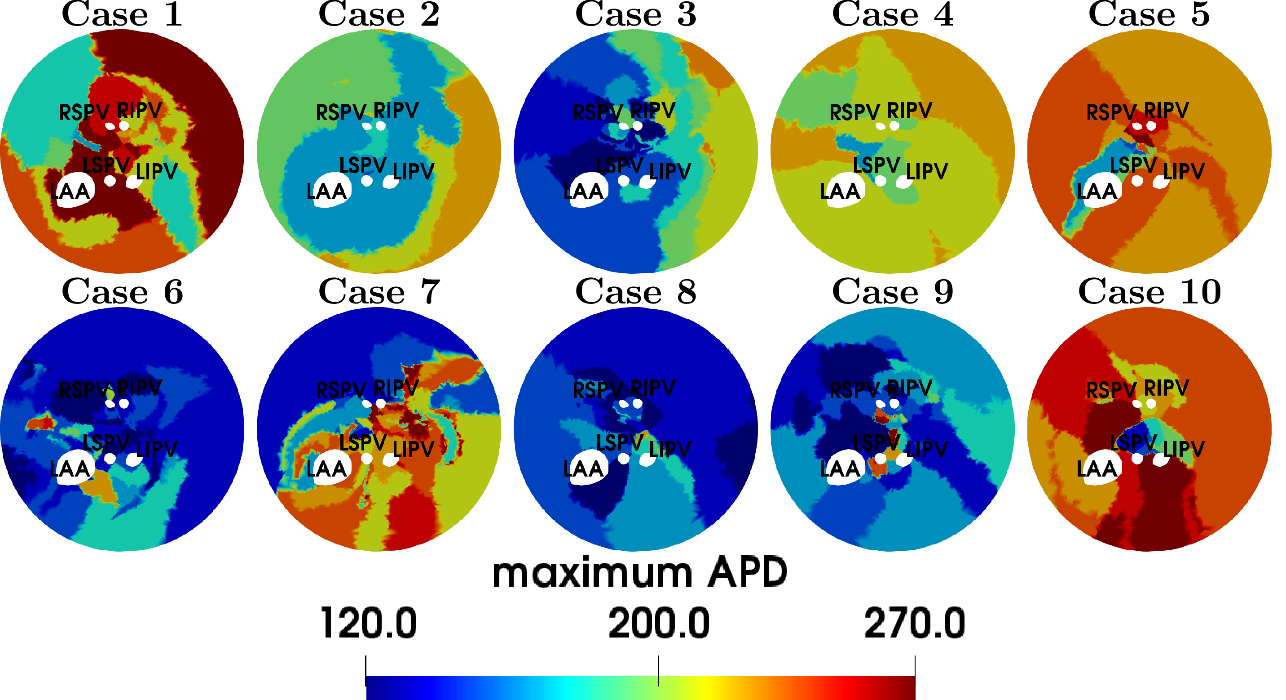


Figure 5 Map of the maximum action potential duration


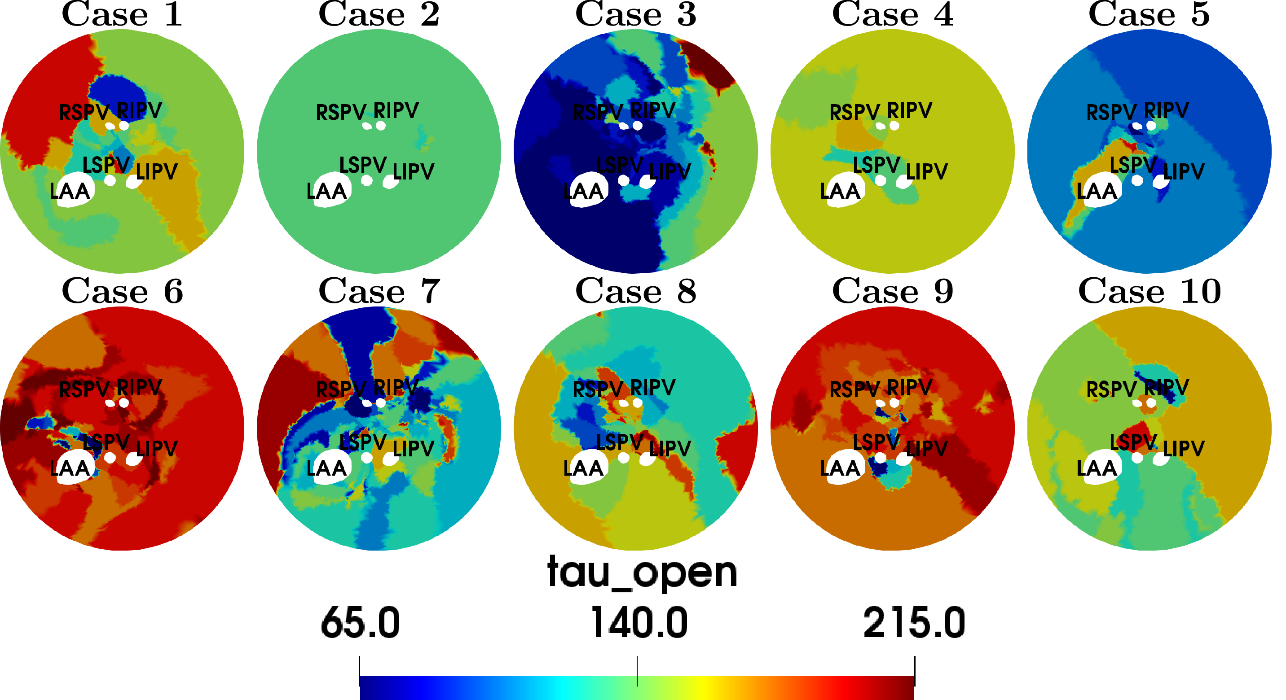


Figure 6 Map of τ_open_


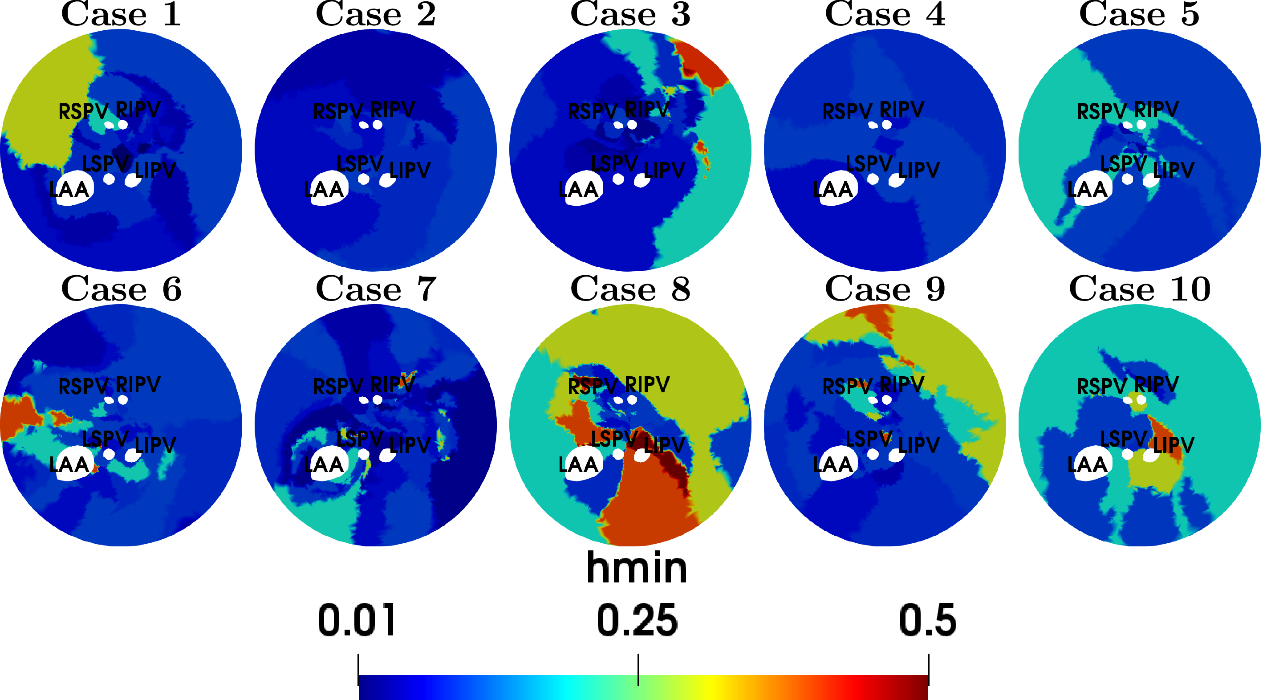


Figure 7 Map of _hmin_


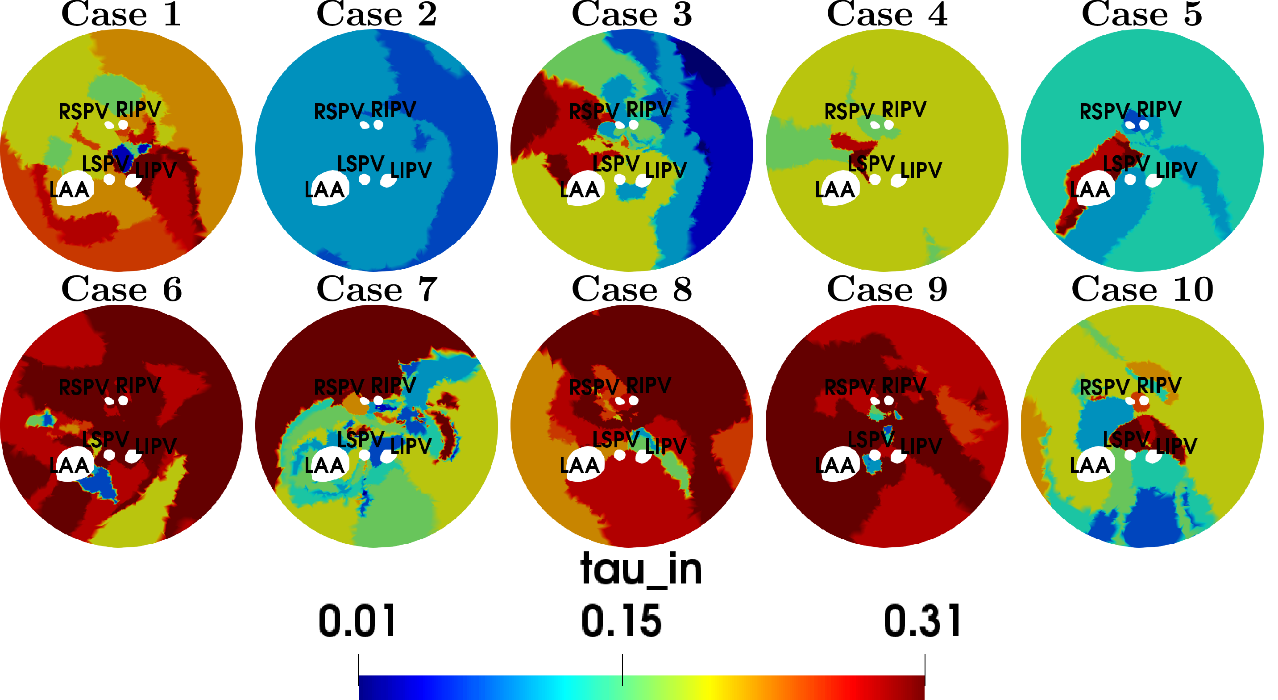


Figure 8 Map of τ_in_

Figure 9 plots the distribution of the model parameters for all of the 10 clinical cases, while in Figure 10 the box plot of the parameters distributions for each patient are presented. Blue boxes refer to cases with no triggering, red boxes to self-terminating AF/AT, green boxes to cases with self-sustaining AF/AT.


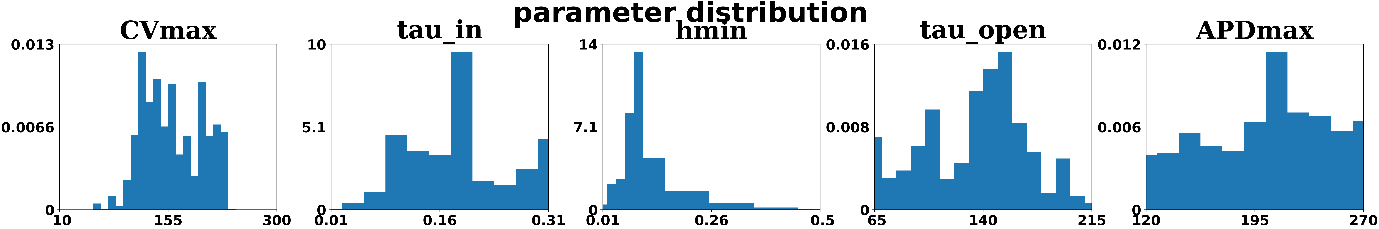


Figure 9 (Histogram) distribution of the model parameters for the whole cohort of 10 cases.


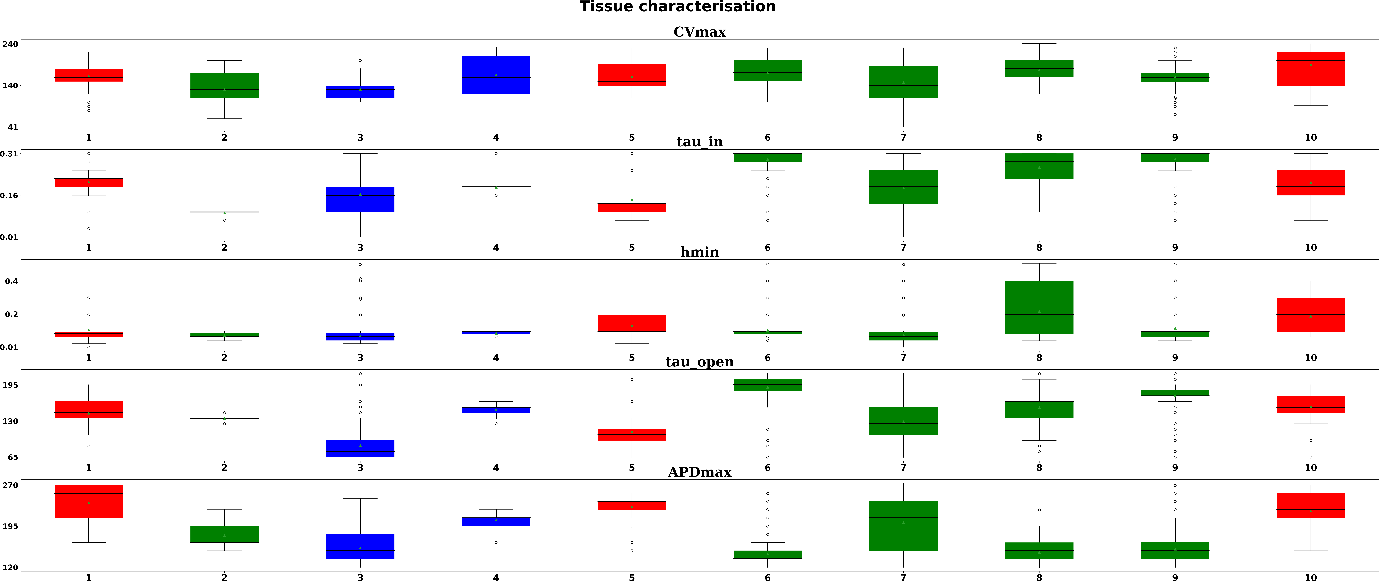


Figure 10 Box plot of the parameters distributions for each patient. Blue boxes refer to cases with no triggering, red boxes to self-terminating AF/AT, green boxes to cases with self-sustaining AF/AT.

## Transient changes in activation dynamics

Figure 11 plots the activation frequency versus time (mean and mean+/-standard deviation) when the stimulus is applied on the left superior pulmonary vein; Figure 12 plots the activation frequency versus time when the stimulus is applied on the left inferior pulmonary vein; Figure 13 plots the activation frequency versus time when the stimulus is applied on the right superior pulmonary vein Figure 14 plots the activation frequency versus time when the stimulus is applied on the right inferior pulmonary vein. Atrial fibrillation (Case 2) and atrial tachycardia with a meandering rotor (Cases 7 and 9) present time variations of the mean activation frequencies, while AF presents a larger standard deviation of the local activation rate if compared to atrial tachycardia. When AT does not present a meander rotor, the local activation rate appears uniform in space and constant in time (Cases 6, 8). As far as self-terminating cases are concerned, we did not report any significant change in frequency before termination.


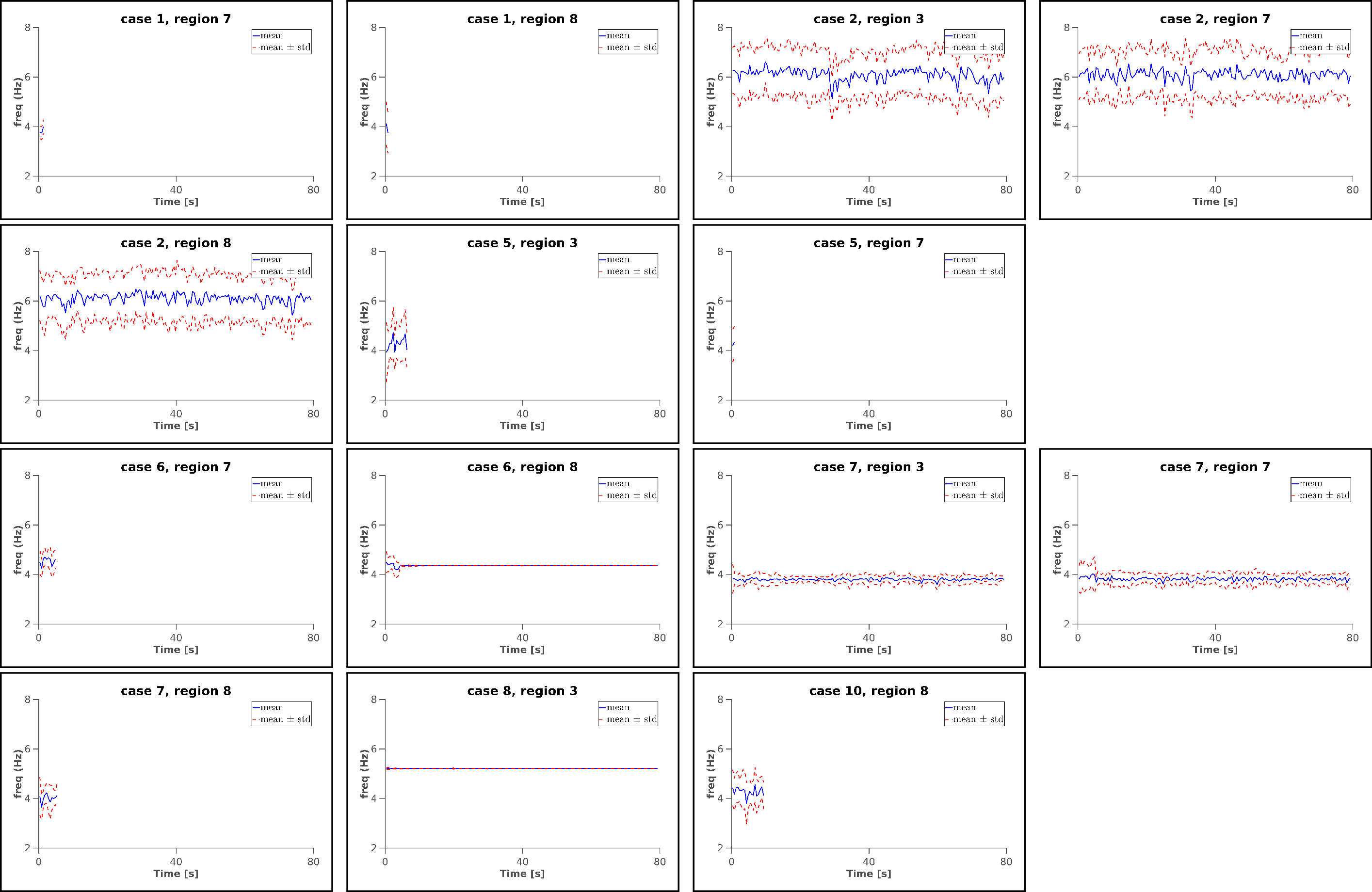


Figure 11 Global activation rate evolution in time for protocol on LSPV


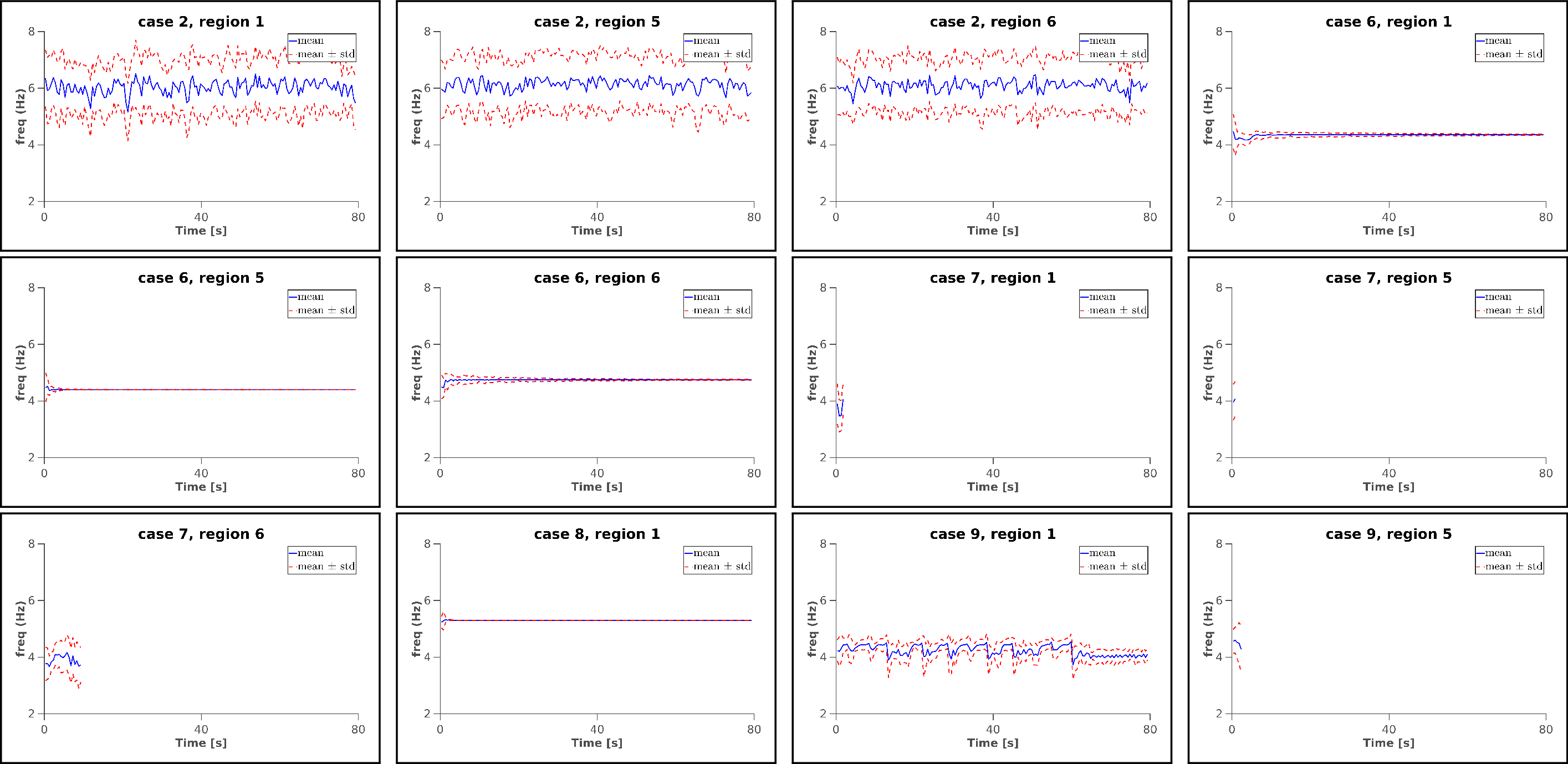


Figure 12 Global activation rate evolution in time for protocol on LIPV


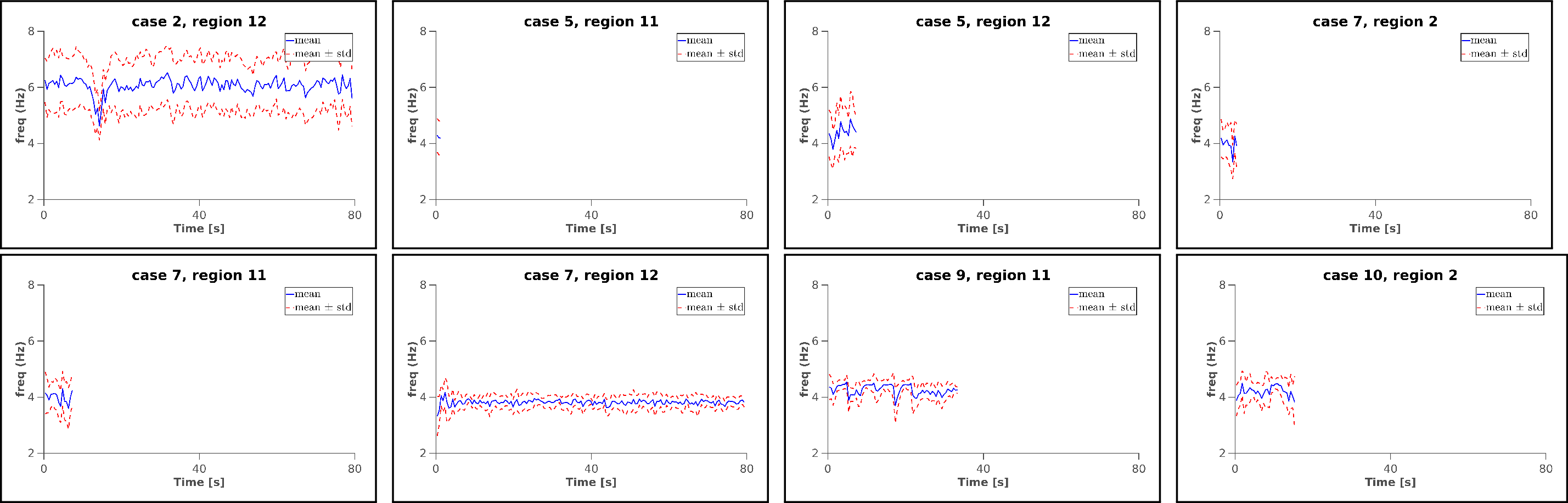


Figure 13 Global activation rate evolution in time for protocol on RSPV


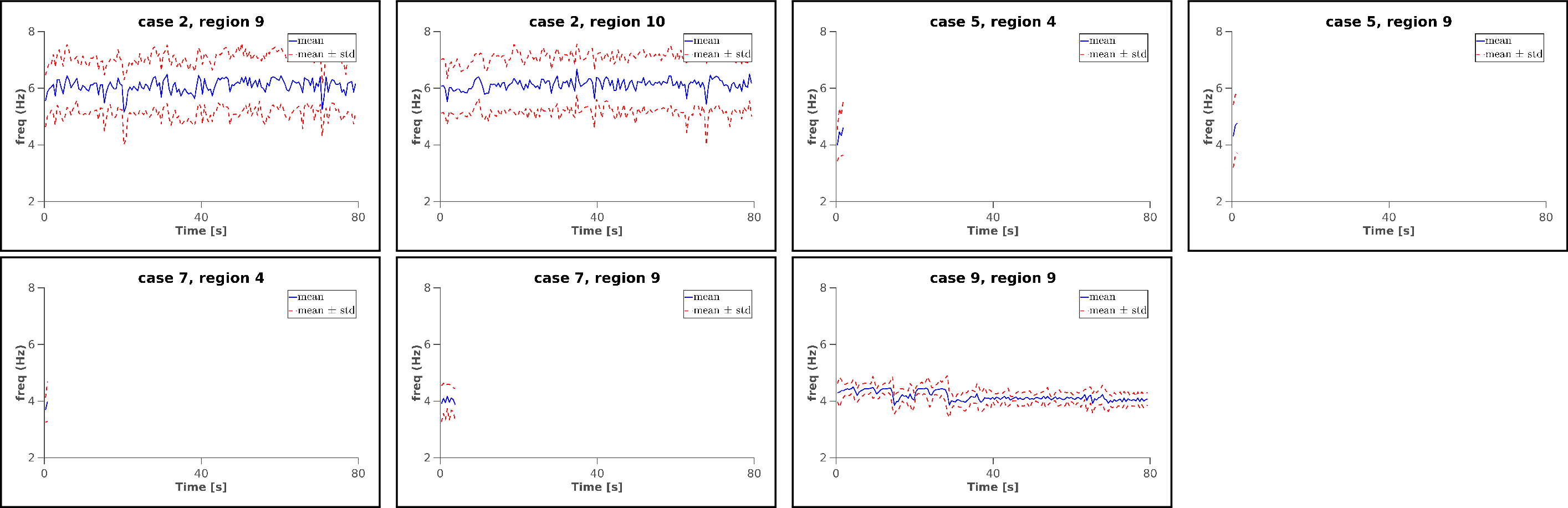


Figure 14 Global activation rate evolution in time for protocol on RIPV

# Phase singularity maps

Figure 15 plots all the combined phase singularity maps for each clinical case.


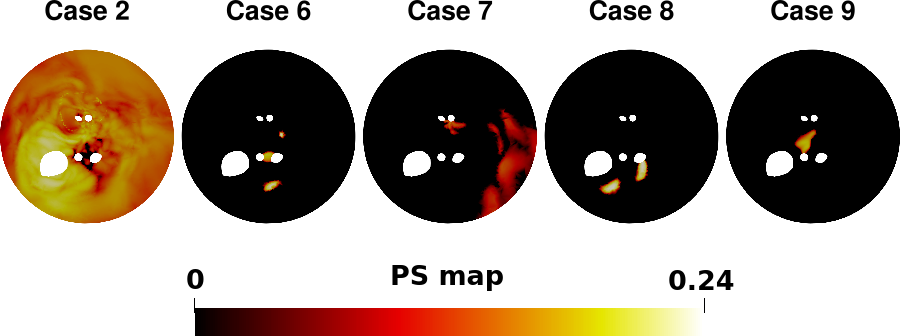


Figure 15 Phase singularity maps for each clinical case. For each clinical case, we plotted on the same surface all the PS densities. Whenever a portion of tissues have 2 or more non-zero PS density value, we assigned the largest one.

Cantwell, C. D., C. H. Roney, F. S. Ng, J. H. Siggers, S. J. Sherwin and N. S. Peters (2015). "Techniques for automated local activation time annotation and conduction velocity estimation in cardiac mapping." Computers in biology and medicine **65**: 229-242.

Corrado, C. and S. A. Niederer (2016). "A two-variable model robust to pacemaker behaviour for the dynamics of the cardiac action potential." Mathematical Biosciences **281**: 46-54.

Corrado, C., J. Whitaker, H. Chubb, S. Williams, M. Wright, J. Gill, M. O. Neill and S. Niederer (2016). Predicting spiral wave stability by personalized electrophysiology models. 2016 Computing in Cardiology Conference (CinC).

Corrado, C., J. Whitaker, H. Chubb, S. Williams, M. Wright, J. Gill, M. D. O’Neill and S. A. Niederer (2017). "Personalized Models of Human Atrial Electrophysiology Derived From Endocardial Electrograms." IEEE Transactions on Biomedical Engineering **64**(4): 735-742.

Corrado, C., S. Williams, R. Karim, G. Plank, M. O’Neill and S. Niederer (2018). "A work flow to build and validate patient specific left atrium electrophysiology models from catheter measurements." Medical Image Analysis **47**: 153-163.

Karim, R., Y. Ma, M. Jang, R. J. Housden, S. E. Williams, Z. Chen, A. Ataollahi, K. Althoefer, C. A. Rinaldi, R. Razavi, M. D. O’Neill, T. Schaeftter and K. S. Rhode (2014). "Surface flattening of the human left atrium and proof-of-concept clinical applications." Computerized Medical Imaging and Graphics **38**(4): 251-266.

Okano, Y., M. Igarashi, H. Sato, S. Fukunaga, K. Takamura, K. Kobayashi and J. Yamasaki (2010). "3-D mapping of Left Atrial Conduction Pattern." Journal of Arrhythmia **26**(3): 170-175.

Rogers, J. M. (2004). "Combined phase singularity and wavefront analysis for optical maps of ventricular fibrillation." IEEE Transactions on Biomedical Engineering **51**(1): 56-65.

Roney, C., M. Beach, A. Mehta, I. Sim, C. Corrado, R. Bendikas, J. A. Solis Lemus, O. Razeghi, J. Whitaker, L. O'Neill, G. Plank, E. Vigmond, S. Williams, M. O'Neill and S. Niederer (2020). "In silico comparison of left atrial ablation techniques that target the anatomical, structural and electrical substrates of atrial fibrillation." Frontiers in Physiology.

Schilling, R. J., N. S. Peters, J. Goldberger, A. H. Kadish and D. W. Davies (2001). "Characterization of the anatomy and conduction velocities of the human right atrial flutter circuit determined by noncontact mapping." Journal of the American College of Cardiology **38**(2): 385-393.

Schroeder, W. J., B. Lorensen and K. Martin (2004). The visualization toolkit: an object-oriented approach to 3D graphics, Kitware.

Weber, F. M., A. Luik, C. Schilling, G. Seemann, M. W. Krueger, C. Lorenz, C. Schmitt and O. Dossel (2011). "Conduction Velocity Restitution of the Human Atrium—An Efficient Measurement Protocol for Clinical Electrophysiological Studies." IEEE Transactions on Biomedical Engineering **58**(9): 2648-2655.

Williams, S. E., N. Linton, L. O'Neill, J. Harrison, J. Whitaker, R. Mukherjee, C. A. Rinaldi, J. Gill, S. Niederer, M. Wright and M. O'Neill (2017). "The effect of activation rate on left atrial bipolar voltage in patients with paroxysmal atrial fibrillation." Journal of Cardiovascular Electrophysiology **28**(9): 1028--1036.

Williams, S. E., L. O’Neill, C. H. Roney, J. Julia, A. Metzner, B. Reißmann, R. K. Mukherjee, I. Sim, J. Whitaker, M. Wright, S. Niederer, C. Sohns and M. O’Neill (2019). "Left atrial effective conducting size predicts atrial fibrillation vulnerability in persistent but not paroxysmal atrial fibrillation." Journal of Cardiovascular Electrophysiology **30**(9): 1416-1427.
